# Sperm Bundles in the Seminal Vesicles of Sexually Mature *Lasius* Ant Males

**DOI:** 10.1371/journal.pone.0093383

**Published:** 2014-03-26

**Authors:** William E. Burnett, Jürgen Heinze

**Affiliations:** 1 Bitterroot Environmental Services Co., Hamilton, Montana, United States of America; 2 Biologie I, University of Regensburg, Regensburg, Germany; Clermont-Ferrand Univ., France

## Abstract

In many insects, sperm cells are produced in bundles with their heads being held together by a glycoprotein matrix secreted by a cyst cell. Mature sperm cells in the seminal vesicles are usually free, but in sawflies and several other insects, such structures (spermatodesmata) remain intact and sperm cells may be ejaculated as bundles. Here we report the occurrence of spermatodesmata in mature males of the ant *Lasius pallitarsis*. Microscopic investigations of the abdominal contents of males immediately prior to their nuptial flights showed that the anterior ends of numerous sperm cells were embedded in an oval-shaped 20 by 30 micrometer extracellular fibrous cap. Individual sperm ranged in length from 55 to 75 micrometers with an average overall length of 65 micrometers. The bulb-shaped heads of the sperm were relatively small, only about 1.5 micrometers in length and about 1.1 micrometers in diameter. The diameter of the sperm tails was approximately 1 micrometer. Observations of live preparations of the spermatodesmata showed increasingly active undulating wave-like movement of the sperm tails as the slide preparations aged. This appears to be the first case of sperm bundles being present in the seminal vesicles of mature ant males – males that are immediately poised to complete their nuptial mating flight.

## Introduction

Animals differ enormously in morphology and anatomy, but their eggs and sperm cells are sometimes believed to be quite uniform: eggs are egg-shaped and sperm are sperm-shaped, with a smooth, oval shaped head and a long tail. In contrast to this view, sperm morphology is quite diverse, and according to a recent review “sperm cells are the most diverse cell type known” [1], with multiflagellate sperm, tailless discoidal sperm, giant sperm, etc. The meaning of this variation is not fully understood, but it is presumably associated with the diversity of fertilization environment and the degree of sperm competition [1], [2].

In addition to variation in morphology, sperm cells may also aggregate and form physical units (“sperm conjugation”). In many insects, but also a number of other animals, sperm cells developing from the same spermatocyte remain arranged in organized bundles with their anterior ends embedded in an extracellular fibrous cap [1], [2]. Such sperm bundles (“spermatodesmata”) usually disorganize with sexual maturation and the seminal vesicles contain only free spermatozoa, but in a number of taxa well-developed spermatodesmata are regularly found in mature sperm [1], [2].

The peculiar life history of social Hymenoptera (ants, bees, and wasps) with mating early after adult emergence and life-long sperm storage thereafter has recently led to an increased interest also in the details of the transfer, storage, viability, and usage of sperm [3]–[10]. Sperm variability is generally not very pronounced across both social and solitary Hymenoptera, and previous studies have only documented that, as in other insects, the length of sperm tails may differ tremendously among species and even among individuals within a species (e.g., [4], [11], [12]). Spermatodesmata with 2^4^ to 2^9^ sperm cells are regularly found in the seminal vesicles of sawflies (suborder Symphyta) [13]–[15] and bundles or fragments of bundles have been observed also in a few apid bees [16]–[18]. In contrast, studies on spermatogenesis and sperm morphology in other Hymenoptera, including ants [see Table S1], show that sperm bundles generally disintegrate when the males mature and the seminal vesicles only contain free sperm cells [19]–[23].

We present here information on the occurrence of sperm bundles in mature male ants of the genus *Lasius*.

## Methods

Males and workers of *Lasius pallitarsis* (Provancher, 1881) were collected on September 1, 2013 from the outer periphery of a partially rotten stump of a cottonwood tree found in the floodplain of the Bitterroot River southeast of the Town of Darby in western Montana USA. The specimens within the wood matrix were placed in dark plastic containers for transport to and subsequent study in the laboratory. A damp paper towel was placed on top of the collected wood in the container to insure adequate humidity was maintained within the collection container. It was noted that the male ants were just about to leave this nest for their nuptial mating flight just a few days ahead of an advancing cold weather front approaching western Montana from British Columbia. During this stage, testes of *Lasius* and most other ants have degenerated and all sperm are stored in the seminal vesicles [24].

The winged ants were identified as males of *Lasius pallitarsis*, a relatively common ant species that is sometimes found in and under wood. This species of ant often inhabits the wood of dead trees for protection of the colony and forages for food outside the nest collecting honeydew from plant sap-feeding Aphids.

For the laboratory investigations, male ants were placed on ice to calm them. The abdomen from each ant was then severed from the body and teased open into a drop of 0.9% saline on a glass slide. A representative sample of the abdominal contents including the seminal vesicles was then mounted on a clean glass slide, covered with a glass coverslip and examined under the microscope. The preparations were examined with a Zeiss Standard 16 microscope equipped with Phase Contrast (PC) and Hoffmann Modulation Contrast (HMC) optics. The preparations were also examined with a Nikon Diaphot 200 Inverted Microscope equipped with Differential Interference Contrast (DIC) optics. Both still and video images of the microscopic preparations were captured using a Canon 5D Mark II DSLR Camera mounted on the respective instruments. In total, 18 males of *L. pallitarsis* were examined.

Following the observations of the live sperm bundles, a dry heat fixed smear of one of the preparations was also prepared and stained with Gram's Stain. This slide was used to further explore the detailed morphology of 20 sperm bundles and for measurements of individual sperm. Measurements were calibrated using a B&L micrometer slide.

No permits were required for our study.

## Results

**Figure 1:**
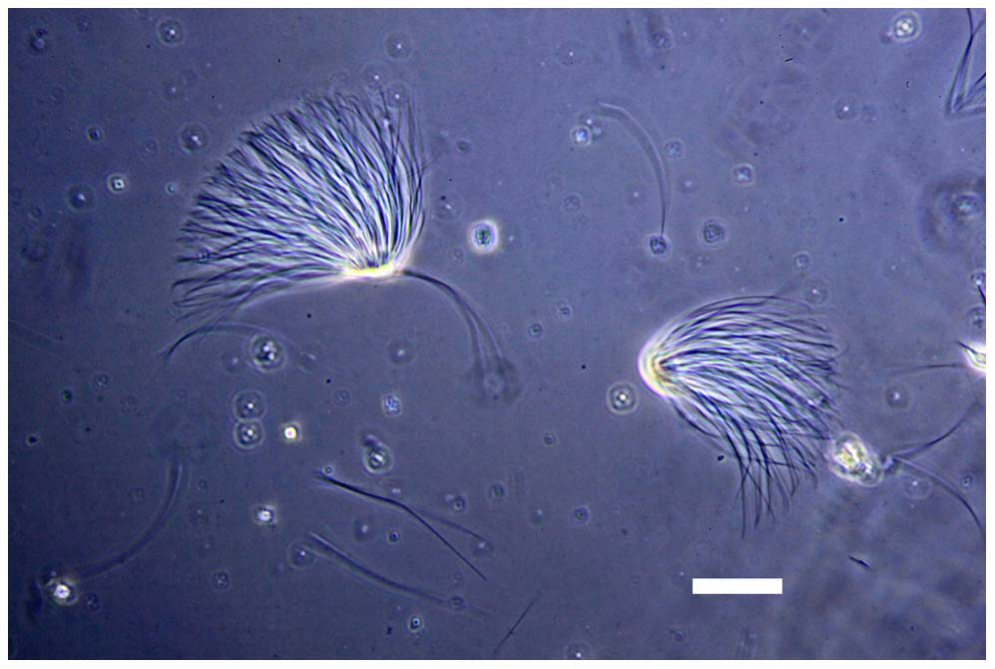
A pair of sperm bundles or spermatodesmata from *Lasius pallitarsis*, Phase Contrast (PC) microscopy. Image captured with blue filter at approximately 400×. Horizontal scale bar represents 20 micrometers.

**Figure 2:**
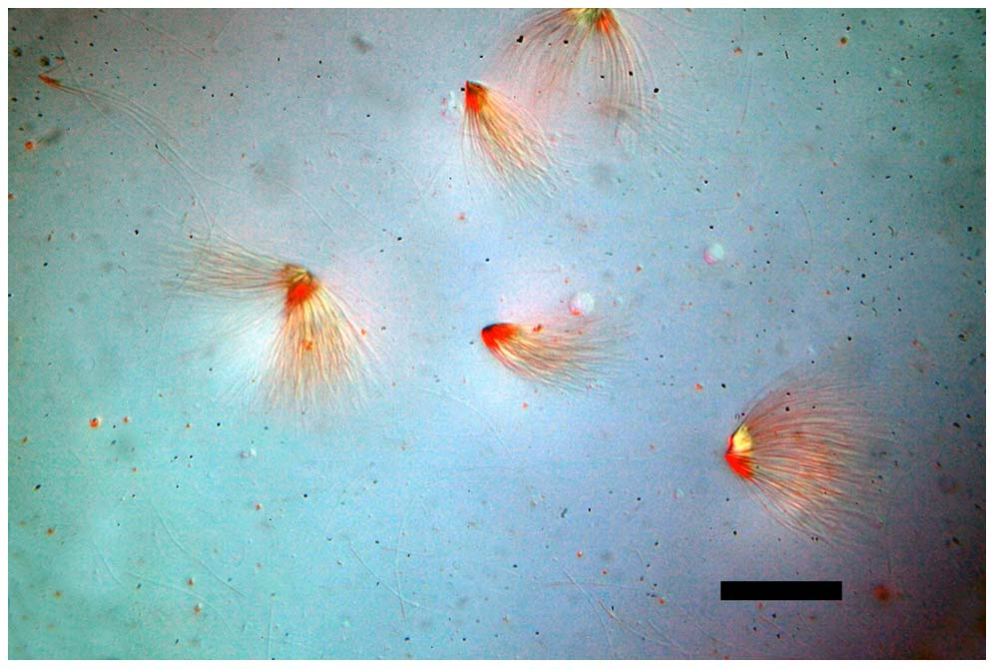
Grouping of sperm bundles or spermatodesmata from *Lasius pallitarsis*, Differential Interference Contrast (DIC) microscopy. Image captured at approximately 200×. Horizontal scale bar represents 50 micrometers.

**Figure 3:**
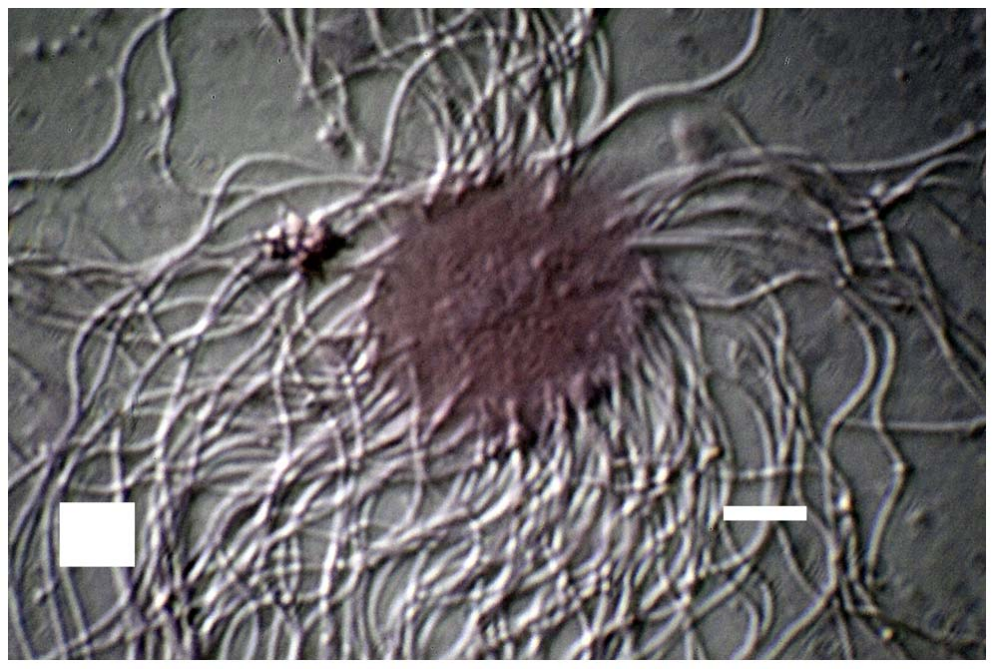
Close up view of *Lasius* sperm embedded in fibrous extracellular cap. Gram's Stain preparation, HMC at approximately 1000× (400× with 2.5× setting on Zeiss Optivar Magnification Changer). Horizontal scale bar represents 10 micrometers.

Microscopy of the abdominal contents of *Lasius* males revealed the presence of both free sperm cells and sperm bundles, which displayed the characteristic morphology of typical insect spermatodesmata with the individual heads of the sperm embedded in an oval-shaped fibrous extracellular cap (Fig. 1, 2, S1), which in contrast to the sperm tails stained red with Gram's Stain (Fig. 3). Although due to the complex intertwined nature of the sperm tails we were unable to precisely measure the number of sperm in each bundle it appeared that the number of per sperm bundle varied.

A digital video was prepared of the sperm bundles as viewed microscopically with PC, HMC and DIC. The video, which we have posted on YouTube, clearly shows the characteristic movement of the sperm tails in the 0.9% saline medium [25]. It was noted that as the live slide preparations aged over time, the individual sperm in the bundles became more active displaying an overall wave-like motion.

The physical teasing of the contents of the male ant's abdomen likely resulted in the breakup of some of the sperm bundles as some free sperm was always visible in both the live and dead preparations. We used these free sperm in the fixed and stained preparation to measure individual sperm length. Individual sperm lengths ranged from 55 micrometers to 70 micrometers, with an overall median of 65 micrometers (n = 10). The bulb-shaped heads of the *Lasius* sperm average only about 1.5 micrometers in length and approximately 1.1 micrometers in diameter. The diameter of the sperm tails was measured at about 1 micrometer (Fig. S2). The oval caps in which the sperm heads were imbedded had a median major axis of 30 micrometers (n = 20; quartiles. 30, 33.5) and a median minor axis of 20 micrometers (n = 20; 18, 22). However, we suspect that the fixing and staining of the preparation may have enlarged (i.e., flattened) the caps, as they appeared to be somewhat larger in the stained preparation than in the live preparations. Also, especially in the live preparations, the cap size appears to be related to the number of attached sperm.

## Discussion

The current observations indicate that sperm of *Lasius pallitarsis* ant males ready to depart for a nuptial flight are in part aggregated in sperm bundles, similar to the spermatodesmata of sawflies and several other animals [1], [2], [13]–[18]. Though our study relies on material only from one colony and thus cannot be generalized, the finding of sperm bundles in the seminal vesicles of males of several related species (S. Aron, M. Pearcy and J.J. Boomsma, pers. comm.) suggests that it is not an idiosyncrasy of a genetically or environmentally stressed colony.

The shape and length of *Lasius* sperm compares closely to the length of individual sperm from fire ants [21], [26], *Pseudomyrmex* [23], and several fungus-growing ants [4]. However, in contrast to other studied ant species [Table S1, Fig. S3], it appears that the bundles in which sperm cells are produced in the testes do not completely disintegrate when males become sexually mature. “Sperm bundles” observed in the accessory testes of males and the spermatheca of freshly mated queens of the leaf-cutter ant *Atta colombica* [12] are not spermatodesmata as reported here for *Lasius.* Instead it appears that fluid dynamics causes the long sperm tails of *A. colombica* to ally with one another so that sperm cells do not migrate individually up the spermathecal duct (J.J. Boomsma and B. Baer, pers. comm.).

Sperm bundles in *Lasius* might result from a non-adaptive, incomplete decomposition of cyst cells after the maturation of sperm cells. Alternatively, the synchronized movement of sperm tails might enhance sperm motility and thus give an advantage in sperm competition. Queens of *Lasius niger* and possibly also other species mate with multiple males [27]–[29], and enhanced motility of sperm might therefore increase the probability of being stored in the spermatheca. Unfortunately we do not have data on the swimming speed of free sperm and sperm bundles in *Lasius* and *s*tudies in other animals do not consistently support motility advantages for conjugated sperm [1], [30], [31]. Future investigations of the complete mating process in *Lasius* will hopefully determine if the queens obtain and store sperm in sperm bundles or as individual sperm and if bundles move faster than free sperm cells.

## Supporting Information

Figure S1 Single sperm bundle or spermatodesm from *Lasius pallitarsis*, Hoffman Modulation Contrast (HMC) microscopy. Image captured at approximately 400×, single frame grab from digital video. Horizontal scale bar represents 20 micrometers.
(TIFF)

Figure S2 Individual *Lasius* sperm. Gram's Stain preparation, HMC at approximately 1000× (400× with 2.5× setting on Zeiss Optivar Magnification Changer). Horizontal scale bar represents 10 micrometers.
(TIFF)

Figure S3 Individual sperm cells of the ant *Cardiocondyla obscurior*. No spermatodesmata are observed in the seminal vesicles of this and many other ant species. Live sperm heads were stained with the fluorescent dye Sybr Green (photo by Alex Schrempf, Univ. Regensburg).
(TIF)

Table S1 Reports on sperm in the seminal vesicles of ants. Though numerous studies report on sperm in the spermatheca of queens or when transferred in spermatophores, only few publications describe spermatogenesis and sperm storage in the seminal vesicles. None of these describes spermatodesmata as seen in the present study in *Lasius pallitarsis*.
(DOCX)
