# Supplementary figures and images for: Sperm Bundles in the Seminal Vesicles of Sexually Mature Lasius Ant Males
Source: PLoS One. 2014 Mar 26;9(3):e93383. doi: 10.1371/journal.pone.0093383 (PMC3966874; doi:10.1371/journal.pone.0093383)

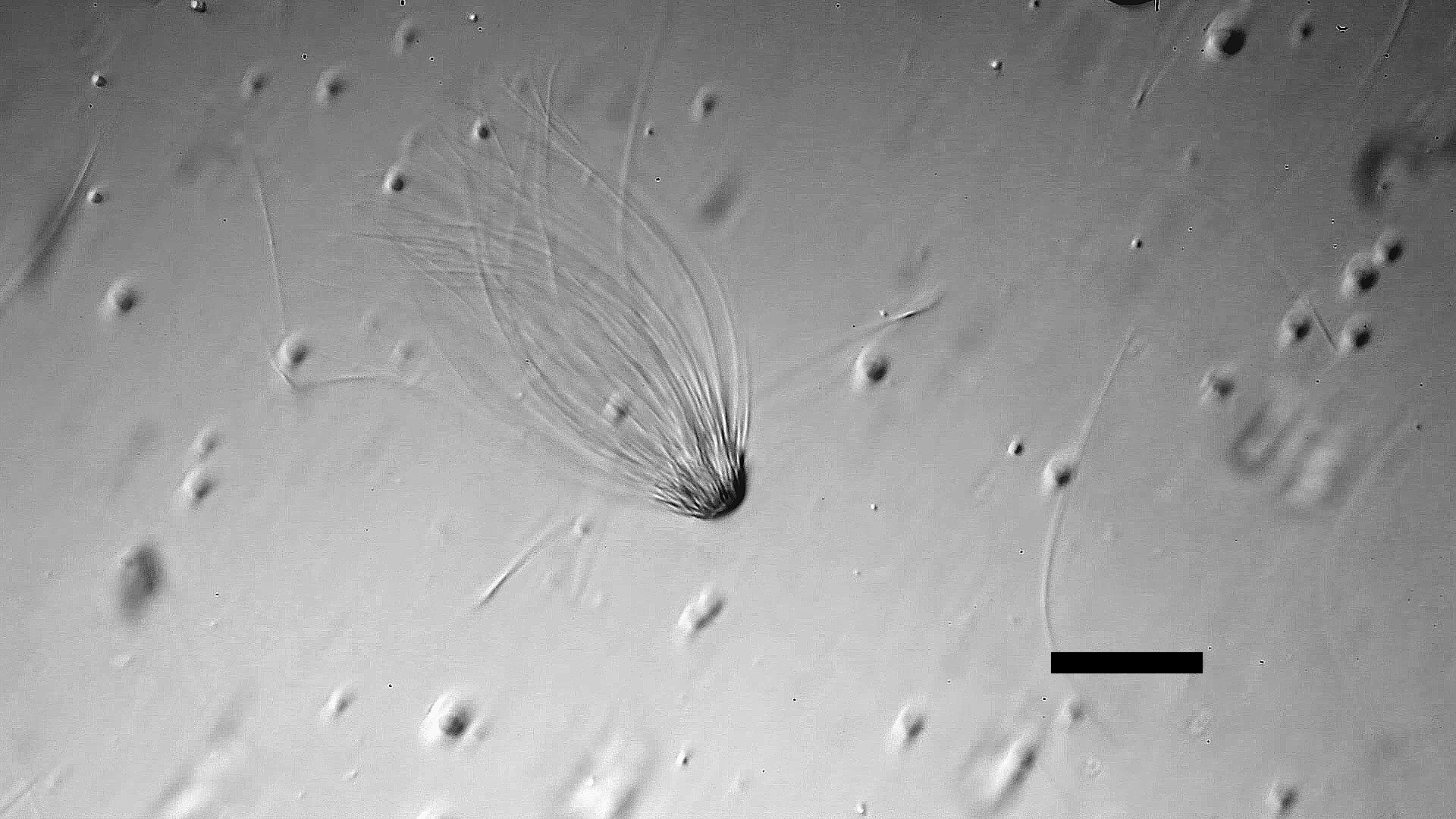

Supplement: Figure S1 — Single sperm bundle or spermatodesm from Lasius pallitarsis, Hoffman Modulation Contrast (HMC) microscopy. Image captured at approximately 400×, single frame grab from digital video. Horizontal scale bar represents 20 micrometers. (TIFF) [file pone.0093383.s001.tiff]

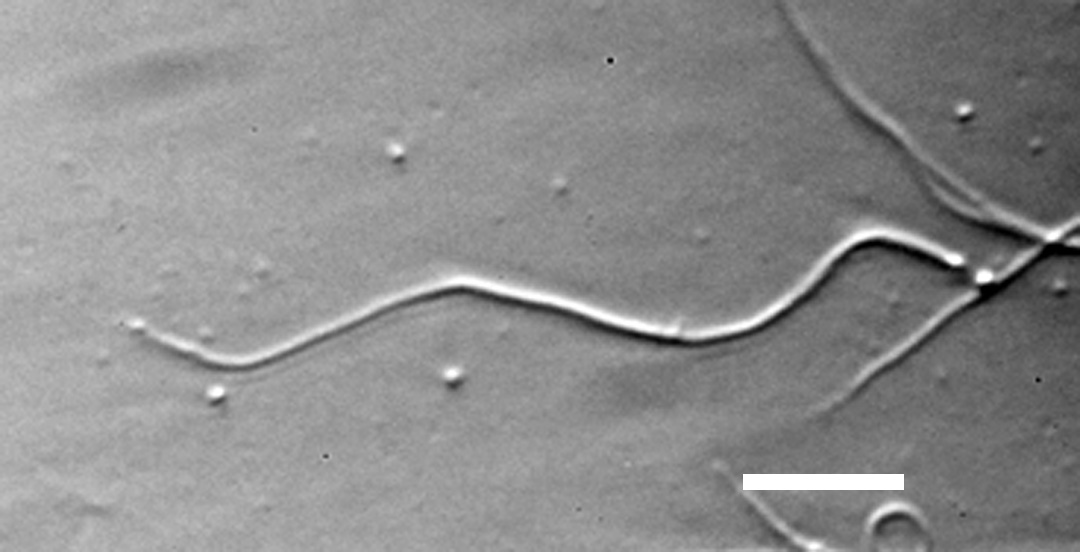

Supplement: Figure S2 — Individual Lasius sperm. Gram's Stain preparation, HMC at approximately 1000× (400× with 2.5× setting on Zeiss Optivar Magnification Changer). Horizontal scale bar represents 10 micrometers. (TIFF) [file pone.0093383.s002.tiff]

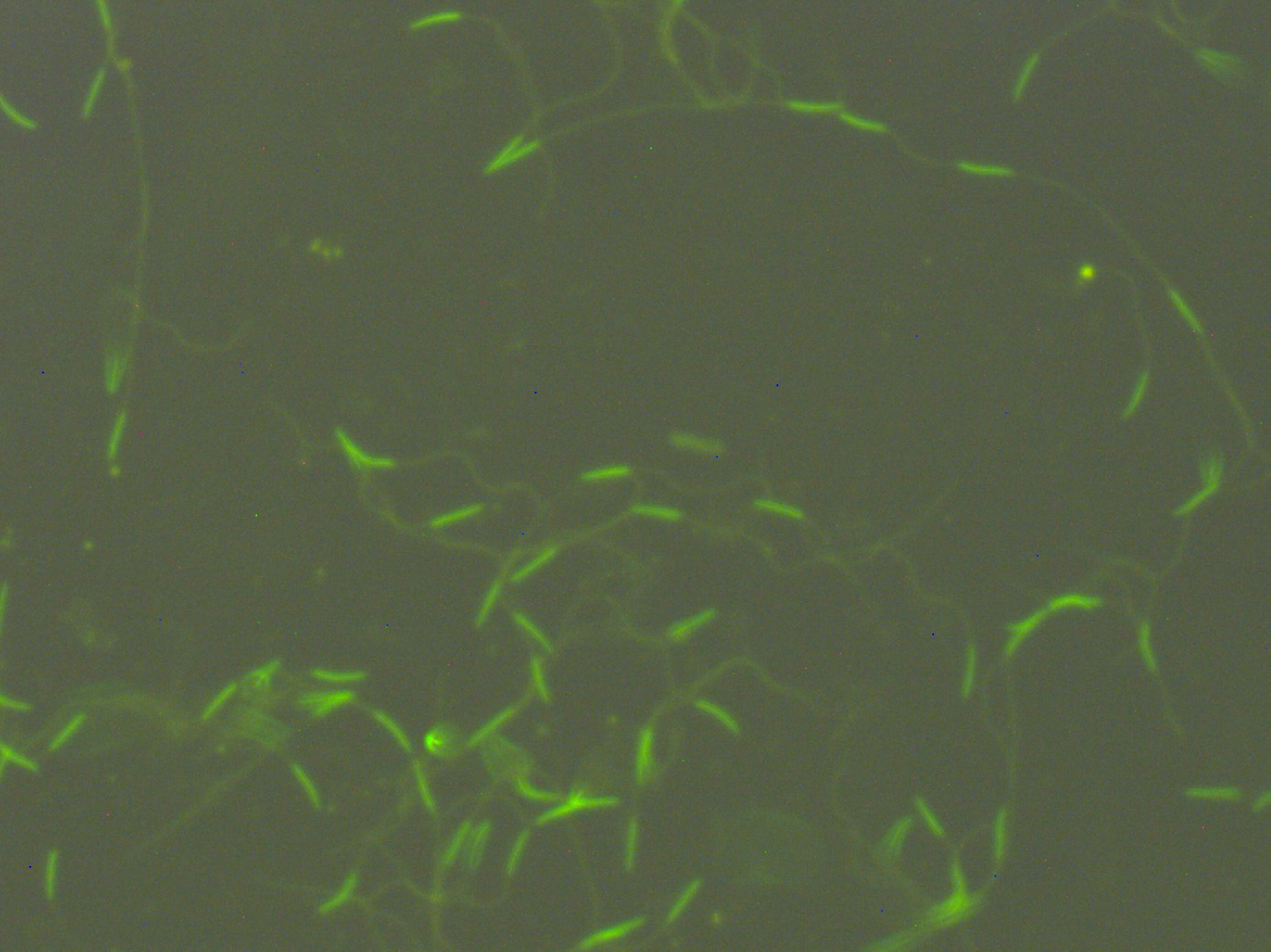

Supplement: Figure S3 — Individual sperm cells of the ant Cardiocondyla obscurior. No spermatodesmata are observed in the seminal vesicles of this and many other ant species. Live sperm heads were stained with the fluorescent dye Sybr Green (photo by Alex Schrempf, Univ. Regensburg). (TIF) [file pone.0093383.s003.tif]
