# Supplementary material for: Sperm Bundles in the Seminal Vesicles of Sexually Mature Lasius Ant Males
Source: PLoS One. 2014 Mar 26;9(3):e93383. doi: 10.1371/journal.pone.0093383 (PMC3966874; doi:10.1371/journal.pone.0093383)
Supplement: Table S1 — Reports on sperm in the seminal vesicles of ants. Though numerous studies report on sperm in the spermatheca of queens or when transferred in spermatophores, only few publications describe spermatogenesis and sperm storage in the seminal vesicles. None of these describes spermatodesmata as seen in the present study in Lasius pallitarsis. (DOCX) [file pone.0093383.s004.docx]

SUPPLEMENTARY TABLE

Table S1: Reports on sperm in the seminal vesicles of ants. Though numerous studies report on sperm in the spermatheca of queens or when transferred in spermatophores, only few publications describe spermatogenesis and sperm storage in the seminal vesicles. None of these describes spermatodesmata as seen in the present study in *Lasius pallitarsis*.

| **Species** | **Reference** |
| --- | --- |
| **No spermatodesmata observed** | |
| **Formicinae** |  |
| *Plagiolepis xene* | 32 |
| *Camponotus* spp. | 20 |
| **Myrmicinae** |  |
| *Anergates atratulus* | 32 |
| Attini, various genera | 12, 22, 33 |
| *Cardiocondyla* spp. | J. Heinze, A. Schrempf unpubl., Fig. S3 |
| *Leptothorax gredleri* | 34 |
| *Myrmoxenus algerianus* | 32 |
| *Pogonomyrmex occidentalis* | 35 |
| *Solenopsis* spp. | 21, 36, 37 |
| **Ponerinae** |  |
| *Hypoponera* spp. | 32 |
| *Gnamptogenys bicolor* | 38 |
| **Pseudomyrmecinae** |  |
| *Pseudomyrmex* spp. | 23 |

32. Heinze J (2000) Testes degeneration and limited sperm supply in ant males with intranidal mating. Mitt Dtsch Ges allg angew Entomol 12: 207-210.

33. Baer B, Dijkstra MB, Mueller UG, Nash, DR, Boomsma JJ (2009) Sperm length evolution in the fungus-growing ants. Behav Ecol 20: 38–45.

34. Oppelt A, Heinze J (2007) Dynamics of sperm transfer in the ant *Leptothorax gredleri*. Naturwissenschaften 94: 781-786.

35. Wiernasz DC, Sater AK, Abell AJ, Cole BJ (2001) Male size, sperm transfer, and colony fitness in the western harvester ant, *Pogonomyrmex occidentalis*. Evolution 55: 324-329.

36. Glancey BM, Lofgren CS (1985) Spermatozoon counts in males and inseminated queens of the imported fire ants, *Solenopsis invicta*  and *Solenopsis richteri* (Hymenoptera: Formicidae). Fla Entomol 68: 162-168.

37. Thompson TE, Blum MS (1967) Structure and behavior of spermatozoa of the fire ant *Solenopsis saevissima* (Hymenoptera: Formicidae). Ann Entomol Soc Am 60: 632-642.

38. Allard D, Ito F, Aikawa Y, Gotoh A, Billen J ( 2011) Testes degeneration in ants: a histological study of *Gnamptogenys bicolor*. Acta Zool 92: 372–376.
